# Current state of social media utilization in neurosurgery amongst European Association of Neurosurgical Societies (EANS) member countries

**DOI:** 10.1007/s00701-021-04939-4

**Published:** 2021-07-27

**Authors:** Aria Nouri, Julien Haemmerli, Alexandre Lavé, Pia Vayssiere, Paul Constanthin, Abdullah Al-Awadhi, Gregory Zegarek, Insa Janssen, Hans Clusmann, Christian F. Freyschlag, Johannes Goldberg, Marcus Czabanka, Martin N. Stienen, Philippe Bijlenga, Karl Schaller

**Affiliations:** 1 Department of Neurosurgery, Geneva University Hospitals, Rue Gabrielle-Perret-Gentil 4, 1205 Geneva, Switzerland; 2 University of Geneva, Geneva, Switzerland; 3 Department of Neurosurgery, RWTH Aachen University Hospital, Aachen, Germany; 4 Department of Neurosurgery, Medical University of Innsbruck, Innsbruck, Austria; 5 Department of Neurosurgery, University Clinic for Neurosurgery, Inselspital, Bern, Switzerland; 6 Department of Neurosurgery, Charité University Hospital, Berlin, Germany; 7 Department of Neurosurgery, Kantonsspital St. Gallen, St. Gallen, Switzerland

**Keywords:** Twitter, Instagram, Facebook, Followers, Public engagement, Social media (SoMe), YouTube, LinkedIn

## Abstract

**Background:**

Social Media (SoMe) is becoming increasingly used in the medical community, and its use has been related with academic productivity. However, utilization of SoMe in the European neurosurgical community has not been assessed systematically.

**Methods:**

An online search was undertaken to discover SoMe accounts of (1) national and related neurosurgical societies listed on the EANS website, (2) neurosurgical journals present on EANS website, (3) neurosurgery centers within EANS member countries, as listed on their website. SoMe accounts of Facebook, Twitter, YouTube, and Instagram were searched for journals and societies, and Twitter, Instagram, and Facebook for neurosurgery departments. The number of likes/followers/subscribers was recorded.

**Results:**

Five (31%) neurosurgery journals had a SoMe presence. The highest number of followers, likes, and tweets was found for *JNNP*, and *Journal of Neurological Surgery Part B* had the most subscribers and video views. SoMe usage was identified for 11 national (28.2%) and 2 multi-national neurosurgical societies. From these, the French Society of Neurosurgery had the largest number of Facebook followers (> 2800) and Likes (> 2700), the Society of British Neurological Surgeons had the largest number of Twitter followers (> 2850), whereas EANS overall had the most followers on Twitter > 5100 and Facebook > 5450. A total of 87 SoMe neurosurgery center accounts were found on either Facebook, Instagram or Twitter, for 64 of 1000 centers (6.4%) in 22 of 40 different countries (55%). Of these 67% (n = 43/64) arose from 6 countries (England, Germany, Italy, Romania, Turkey, Ukraine). There were more Facebook accounts (n = 42) than Instagram accounts (n = 23) or Twitter accounts (n = 22).

**Conclusion:**

SoMe use amongst neurosurgical societies and departments in Europe is very limited. From our perspective, explanations are lacking for the correlated numbers to the market shares of SoMe in the respective countries. Further research, including a survey, to follow up on this important topic should be undertaken among EANS members.

## Introduction

Social media (SoMe) has been developed to connect individuals and groups of similar interest through online communication platforms. Based on World Bank data from 2017, it was estimated that nearly 50% of the world population was using the internet [19]. Recently, Facebook reported (March 31, 2020) that monthly active users were 2.60 billion [6], Twitter reported (Q1, 2020) that monetizable daily active users amounted to 166 million [15]. YouTube states on their website that “2 billion logged-in users visit YouTube each month, and every day people watch over a billion hours of video and generate billions of views” [21], Instagram has around 1 billion active users (as of January 2019 [7], and LinkedIn has over 740 million members [8].

SoMe evolved from chatgroups for adolescents and crude ideas of sharing information and pictures into highly elaborated communication platforms that reach out to the public for the global sharing of information. As with the increased use of SoMe amongst people and organizations, medical health professionals and societies are increasingly adopting these platforms to engage with the general public as well as target audiences [9, 14, 18]. For medical communities, SoMe is used to share scientific publications or breakthroughs or current positions on important topics in the specialized field.

Multiple publications from different medical specialties have highlighted the increased awareness of the medical community to the benefits of SoMe engagement. Indeed, it has been shown that SoMe engagement is related to citations, downloads, Altmetric scores, and Mendeley reads [5, 10, 12], and it has been specifically reported to be related with academic bibliometric profiles in neurosurgery [3].

The current use of SoMe in Neurosurgery in North America was previously reported, showing that Facebook and Twitter are the most actively used platforms and that private practices dominate the SoMe presence [2]. Recently, it was also shown that there are numerous neurosurgery related Instagram accounts posting images and videos and having a combined following of over 600,000 accounts [20]. However, the use of SoMe in neurosurgery and subsequently its reach are well known in North America, whereas its use in other parts of the world including Europe remains poorly studied. In order to better understand the current landscape of SoMe use in European Neurosurgery, this study sought to investigate the usage of SoMe platforms by neurosurgical centers, journals and neurosurgical organizations amongst member states of the European Association of Neurosurgical Societies (EANS). The aim of the study is to provide an overview of the current state of social media utilization amongst EANS member country institutions.

## Methods

We focused our search strategies on states within the EANS. Neurosurgical journals investigated included those listed on the EANS website and originating in a member state (https://www.eans.org/page/European_Neurosurgical_Journals). Likewise, European neurosurgical society included those based on the listing of European Member Societies on the EANS website (https://www.eans.org/general/custom.asp?page=societies). In addition to reviewing individual society SoMe usage, multi-national societies within Europe were investigated for SoMe utilization by searching member societies of the World Federation of Neurosurgical Societies (WFNS) (https://www.wfns.org/all-member-societies). For all inquiries, websites were initially screened to find potential SoMe links. Thereafter, the journal or organizational names and abbreviations were searched in the following platforms: Facebook, Twitter, Instagram, and YouTube (search was conducted in June 2020).

Neurosurgery departments within the EANS member countries registered online with EANS (www.eans.org/page/European_Neurosurgical_Centers) were searched systematically by official institution name and/or their abbreviations for a SoMe account (Twitter, Facebook, and Instagram), and the number of followers/likes/tweets/posts was recorded. The search was conducted with the Google search engine and with the search function of the respective SoMe platforms. Keywords included the English and native language names (where applicable), in addition to the SoMe platform name (search was conducted in May/June, 2021).

For comparison of SoMe activity in North America, the social media activity was recorded for the Congress of Neurological Surgeons (CNS) and the American Association of Neurological Surgeons (AANS), retrieved in Dec., 2020.

For all variables (likes, tweets, followers, etc.) numbers of less than 50 where designated as < 50. Numbers greater than 50 were rounded down to increments of 50 and designated with a greater than sign (for example 2069 was designated > 2050, 320 was designated > 300).

## Results

### Neurosurgery journals

A total of 16 neurosurgery specific journals were reviewed, including 15 present on the EANS website as well as *Acta Neurochirurgica* (the EANS affiliated journal) (Table 1). Five of those journals (31%) had a presence on either Twitter, Facebook, Instagram, or YouTube. None of these were present in all four platforms, but *Journal of Neurology, Neurosurgery and Psychiatry (JNNP)* and Turkish Neurosurgery had a presence in 3 platforms each. Twitter and Facebook were each used by 3 journals and Instagram and YouTube by 2 journals. The highest number of followers, likes, and tweets was reported for *JNNP*, and *Journal of Neurological Surgery Part B (Skull Base)* had the most subscribers and video views.

**Table 1:** European Neurosurgical Journals as listen by EANS and their current social media utilization

Journal Name	Twitter		Facebook		Instagram		Youtube	
	**Followers**	**Tweets**	**Followers**	**Likes**	**Followers**	**Posts**	**Subscribers**	**Views**
***Acta Neurochirurgica***	> 1950	> 400	x	x	x	x	x	x
*British Journal of Neurosurgery*	x	x	x	x	x	x	x	x
*Burdenko's Journal of Neurosurgery*	x	x	x	x	x	x	x	x
***Journal of Neurology, Neurosurgery and Psychiatry***	> 10,000	> 15,900	> 23,350	> 22,600	x	x	> 950	> 10,250
***Journal of Neurological Sciences***	x	x	> 950	> 900	x	x	x	x
*Journal of Neurological Surgery Part A*	x	x	x	x	x	x	x	x
***Journal of Neurological Surgery Part B***	x	x	x	x	> 2150	> 50	> 1850	> 89,250
*Neurocirugia*	x	x	x	x	x	x	x	x
*Neurochirurgie*	x	x	x	x	x	x	x	x
*Neurosurgical Review*	x	x	x	x	x	x	x	x
*Polish Journal of Neurology and Neurosurgery*	x	x	x	x	x	x	x	x
*Romanian Neurosurgery*	x	x	x	x	x	x	x	x
*Russian Journal of Neurosurgery*	x	x	x	x	x	x	x	x
*Tijdschrift voor Neurologie en Neurochirurgie*	x	x	x	x	x	x	x	x
***Turkish Neurosurgery***	> 150	> 50	< 50	< 50	> 950	> 50	x	x
*Ukrainian Neurosurgical Journal*	x	x	x	x	x	x	x	x
***By Comparison (retrieved Dec/2020)***								
***Neurosurgery***	> 20,800	> 1300	> 33,900	> 32,900	> 8700	> 700	> 7800	> 1.4 million
***Journal of Neurosurgery (JNS***	> 23,400	> 8100	> 50,600	> 48,400	> 34,900	> 400	x	x

The journals of CNS (*Neurosurgery*) and AANS (*Journal of Neurosurgery*) in comparison had a significant presence and user engagement on multiple SoMe platforms, with the highest use evident on Facebook (> 48,400 followers for JNS and > 32,900 followers for Neurosurgery), and a high number of YouTube videos for CNS videos (> 1.4 million).

### National and multi-national European neurosurgical societies

A total of 39 country societies listed on EANS, and 3 multi-national societies (including EANS) were assessed for SoMe usage (Table 2). SoMe usage was identified for 11 national (28.2%) and 2 multi-national neurosurgical societies. The most commonly used platforms were Facebook (11 accounts, 28.2%) and Twitter (5 accounts 12.8%). There was only 1 Instagram account found (2.6%) and 2 YouTube accounts (5.1%). All societies which had Twitter accounts also had a Facebook account. The French Society of Neurosurgery had the largest number of Facebook followers (> 2800) and Likes (> 2700), followed by the Hellenic Neurosurgical Society with (> 1800 follows, > 1800 Likes). All other Facebook accounts had less than 1000 followers. The Society of British Neurological Surgeons had the largest number of Twitter followers (> 2850) followed by the Spanish Society of Neurosurgery (> 450).

**Table 2:** State and multi-national societies and their current use of social media

	**Twitter**		**Facebook**		**Instagram**		**YouTube**	
	**Followers**	**Tweets**	**Followers**	**Likes**	**Followers**	**Posts**	**Subscribers**	**Views**
**National societies**								
*Albanian Neurosurgical Society*	x	x	x	x	x	x	x	x
*Armenian Neurosurgical Association*	x	x	x	x	x	x	x	x
*Austrian Society of Neurosurgery*	x	x	x	x	x	x	x	x
*Association of Neurosurgeons in Bosnia and Herzegovina*	x	x	x	x	x	x	x	x
*Association of Russian Neurosurgeons*	x	x	x	x	x	x	x	x
*Belgian Society of Neurosurgery*	x	x	x	x	x	x	x	x
*Bulgarian Society of Neurosurgery*	x	x	x	x	x	x	x	x
*Croatian Neurosurgical Society*	x	x	x	x	x	x	x	x
*Cyprus Neurosurgical Society*	x	x	x	x	x	x	x	x
*Czech Neurosurgical Society*	x	x	x	x	x	x	x	x
*Danish Neurosurgical Society*	x	x	x	x	x	x	x	x
*Dutch Association of Neurosurgeons*	x	x	x	x	x	x	x	x
*Estonian Neurosurgical Society*	x	x	x	x	x	x	x	x
*Finnish Neurosurgical Society*	x	x	x	x	x	x	x	x
***French Society of Neurosurgery***	< 100	< 50	> 2800	> 2750	x	x	x	x
*German Society for Neurosurgery*	x	x	X	x	x	x	x	x
***Hellenic Neurosurgical Society***	x	x	> 1800	> 1800	x	x	< 100	> 1400
*Hungarian Neurosurgical Society*	x	x	x	x	x	x	x	x
*Israel Neurosurgical Society*	x	x	x	x	x	x	x	x
***Italian Neurosurgical Society***	x	x	> 250	> 200	x	x	0	> 250
***Kazakh Association of Neurosurgeons***	x	x	> 100	> 100	x	x	x	x
*Kosovo Neurosurgical Society*	x	x	x	x	x	x	x	x
*Latvian Association of Neurosurgeons*	x	x	x	x	x	x	x	x
*Lithuanian Neurosurgical Society*	x	x	x	x	x	x	x	x
*Macedonian Association of Neurosurgeons*	x	x	x	x	x	x	x	x
*Moldova Association of Neurosurgeons*	x	x	x	x	x	x	x	x
*Norwegian Neurosurgical Association*	x	x	x	x	x	x	x	x
***Polish Society of Neurosurgeons***	x	x	< 100	< 50	x	x	x	x
***Portuguese Neurosurgery Society***	< 100	> 50	< 100	< 50	x	x	x	x
***Romania Society of Neurosurgery***	x	x	< 100	< 50	x	x	x	x
*Serbian Neurosurgical Society*	x	x	x	x	x	x	x	x
*Slovak Neurosurgical Society*	x	x	x	x	x	x	x	x
*Slovenian Neurosurgical Society*	x	x	x	x	x	x	x	x
***Society of British Neurological Surgeons***	> 2850	> 300	> 100	> 100	x	x	x	x
***Spanish Society of Neurosurgery***	> 450	> 750	> 350	> 300	x	x	x	x
*Swedish Neurosurgical Society*	x	x	x	x	x	x	x	x
*Swiss Society of Neurosurgery*	x	x	x	x	x	x	x	x
***Turkish Neurosurgical Society***	> 150	> 50	> 850	> 850	> 950	> 50	x	x
***Ukrainian Association of Neurosurgeons***	x	x	> 500	> 450	x	x	x	x
**Multi-national societies**								
***Scandinavian Neurosurgical Society***	x	x	> 100	> 100	x	x	x	x
*Southeast Europe Neurosurgical Society*	x	x	x	x	x	x	x	x
***European Association of Neurosurgical Societies***	> 5100	> 2450	> 5450	> 5300	x	x	x	x

In terms of multi-national societies, EANS utilized both Twitter and Facebook accounts and had the most followers with > 5100 and > 5450, respectively. The Scandinavian Society had a Facebook account. No account associated with Southeast Europe Neurosurgical Society was found.

### EANS neurosurgical centers

At the time of the search, a total of 1000 centers were listed on the EANS website (Table 3). A total of 87 SoMe accounts were found on either Facebook, Instagram, or Twitter, for 64 centers (SoMe use of 6.4%) in 22 different countries (55% of countries, 40 countries). Of these 67% (n = 43/64) arose from 6 countries (England, Germany, Italy, Romania, Turkey, Ukraine). There were more Facebook accounts (n = 42) than Instagram accounts (n = 23) or Twitter accounts (n = 22). England and Turkey had the most EANS affiliated centers with a SoMe presence (n = 9). The Brain Institute in Romania had the largest number of Facebook Followers (> 17,500), and National Center for Neurosurgery in Kazakhstan had the highest number of Instagram followers (20,500), whereas the 2 most active twitter accounts were located in England: King’s College Neurosurgery (> 3600 followers) and The Royal London Hospital Neurosurgery (> 3500). 17 departments used more than one SoMe platform, and 6 used all 3 SoMe platforms. One department, Klinik für Neurochirurgie, UKSH (Universitätsklinikum Schleswig–Holstein Hochleistungsmedizin) had 2 accounts one with slightly different names: 1 Twitter account in English and 1 German account for Instagram.

**Table 3:** EANS neurosurgical centers

Neurosurgical centers	Facebook	Instagram		Twitter	
	**Followers**	**Followers**	**Posts**	**Followers**	**Tweets**
General Hospital Damiaan (Belgium)	> 5200	x	x	x	x
Heart and Brain Center of Clinical Excellence (Bulgaria)	> 500	x	x	x	x
Clinical Hospital KB Dubrava (Croatia)	> 650	x	x	x	x
University Hospital Kralovske Vinohrady (Czech Republic)	> 850	x	x	x	x
RLH Neurosurgery (England)	x	x	x	> 3500	> 400
UHB Neurosurgery (England)	x	x	x	> 600	> 100
King’s Neurosurgery (England)	> 1300	> 350	> 50	> 3600	> 1500
Preston Neurosurgery (England)	x	x	x	> 600	> 150
UHP Neurosurgery (England)	x	x	x	> 100	< 50
Leeds Neurosurgery (England)	> 500	x	x	> 1000	> 700
St. George’s Neuro (England)	> 300	x	x	> 300	> 100
Great Ormond Street Neurosurgery (England)	x	x	x	> 1400	> 200
Wessex Neurological Centre, Department of Neurosurgery (England)	> 100	x	x	> 400	> 200
International Neuroscience Institute (INI), Hanover (Germany)	> 250	x	x	x	x
University Hospital and Faculty of Medicine, Eberhard Karls University Tübingen (Germany)	< 50	x	x	x	x
Klinik für Neurochirurgie UKSH (Germany)*	x	> 100	< 50	x	x
Department of Neurosurgery UKSH (Germany)*	x	x	x	> 50	< 50
Neurosurgery, TU Munich (Germany)	x	x	x	> 400	> 150
Neurochirurgie-Universitätsmedizin Göttingen (Germany)	x	x	x	> 50	< 50
General Military Hospital of Thessaloniki (Greece)	> 750	x	x	x	x
Szent János Hospital (Hungary)	> 200	x	x	x	x
Neurosurgery Budapest (Hungary)	> 200	> 1000	< 50	x	x
University Hospital Policlinico Paolo Giaccone (Italy)	> 1300	x	x	x	x
Policlinico A. Gemelli, Roma (Italy)	> 2500	x	x	x	x
Policlinico Tor Vergata, Roma (Italy)	< 50	x	x	x	x
Neurosurgery Division, Dip. Neuroscience and Rehabilitation, Arcispedale S. Anna Cona, Ferrara (Italy)	x	> 100	< 50	x	x
U.O.C. Neurosurgery, Hospital S. Maria Goretti, Latina (Italy)	x	> 100	< 50	x	x
Mater Olbia Hospital–Qatar Foundation Endowment and Gemelli Foundation (Italy)	x	> 50	> 200	x	x
Hospital Santo Spirito (Italy)	x	> 100	< 50	x	x
Verona (Italy)	x	< 50	< 50	x	x
Hospital Uni. Del Rocio (Italy)	x	> 200	< 50	> 400	> 100
National Center for Neurosurgery (Kazakhstan)	> 3800	> 20,500	> 500	> 150	> 800
Institute Of Neurology and Neurosurgery (Moldova)	> 3700	> 100	< 50	x	x
Clinical Republican Hospital (Moldova)	> 1400	x	x	x	x
Department of Public Neurosurgery, Independent Healthcare Center, Voivodship Medical Center (Poland)	> 1300	x	x	x	x
Emergency Clinical Hospital “Bagdasar-Arseni”, Bucuresti I (Romania)	> 350	x	x	x	x
Neurohope (Romania)	> 3800	> 200	< 50	> 50	< 50
Brain Institute (Romania)	> 17,500	> 800	< 100	< 50	> 350
County Emergency Hospital, Tg. Mures (Romania)	> 600	x	x	x	x
County Emergency Hospital “Pius Brinzeu”, Timisoara (Romania)	> 2400	x	x	x	x
National Medical Research Center for Neurosurgery named after Academician N.N. Burdenko (Russia)	> 2700	> 7300	> 300	< 50	> 150
Federal State Budgetary Institution, Federal Neurosurgical Center (Russia)	> 350	x	x	x	x
Centro Medico Asturias (Spain)	> 1600	> 450	> 100	> 350	> 550
Neurosurgical Clinic, Skåne University Hospital (Sweden)	< 50	x	x	x	x
Neurochirurgie LUMC (The Netherlands)	x	x	x	> 50	< 50
Neurologie/Neurochirurgie UMCU (The Netherlands)	x	> 700	< 100	x	x
Lokman Hekim University, Faculty of Medicine, Department of Neurosurgery (Turkey)	< 50	> 2200	> 200	x	x
Bursa Acibadem Hospital, Department of Neurosurgery (Turkey)	< 50	x	x	x	x
Pamukkale University Hospitals, Department of Neurosurgery (Turkey)	< 50	> 1700	> 150	x	x
Eskisehir State Hospital, Department of Neurosurgery (Turkey)	> 300	x	x	x	x
Haydarpasa Numune Training and Research Hospital, Department of Neurosurgery (Turkey)	< 50	x	x	x	x
Vm Medical Park Samsun Hastanesi (Turkey)	> 2500	x	x	x	x
Istanbul Medeniyet University, Faculty of Medicine, Department of Neurosurgery (Turkey)	x	> 450	> 200	x	x
Osmangazi University, Department of Neurosurgery (Turkey)	x	> 200	> 100	x	x
ITF Beyin ve Sinir Cerrahisi (Turkey)	x	> 500	< 50	x	x
Dnipro Regional Clinical Hospital Named After I.I.Mechnikov Neurosurgical Department (Ukraine)	> 1200	x	x	x	x
Kharkiv City Clinical Hospital №7, Neurosurgical Department (Ukraine)	> 1200	> 100	< 50	x	x
Romodanov Neurosurgery Institute, National Academy of Medical Sciences of Ukraine, Neurosurgical Department (Ukraine)	> 2800	x	x	x	x
Kyiv City Center for Pediatric Neurosurgery (Ukraine)	> 450	x	x	x	x
International Neurosurgical Center (Ukraine)	> 5700	x	x	x	x
Odessa Regional Clinical Hospital, Neurosurgical Department (Ukraine)	< 50	x	x	x	x
MSN Neurosurgery (Scotland)	x	x	x	> 150	< 50
INS Neurosurgery (Scotland)	x	x	x	> 1400	> 950
Geneva Neurosurgery (Switzerland)	x	> 350	< 50	> 250	< 100
University Hospital of Wales, Department of Neurosurgery (Wales)	> 50	x	x	x	x
Total	42	23		22	

*Same clinical department (accounts in different language)

## Discussion

### The state of SoMe in neurosurgery in Europe

The results of our research show that European SoMe use from an institutional standpoint remains low, with only 6.4% of neurosurgical centers listed on the EANS website having a SoMe presence. Furthermore, while SoMe presence occurred amongst approximately half (55%) of the affiliated countries, two-thirds of SoMe accounts originated from only 6 countries. Part of the reason for this discrepancy is possibly due to the fact that the English language dominates many of the platforms such as Twitter [4]. This is supported by the fact that the 2 most followed neurosurgery center Twitter accounts (King’s Neurosurgery and RLH Neurosurgery), the most followed national society (Society of British Neurological Surgeons), and the most followed journal (*JNNP*) all originate in England. However, strong SoMe engagement was also seen sporadically in other European countries. For example, the Brain Institute in Romania had a following of over 17,500 on Facebook, and the National Center for Neurosurgery in Kazakhstan had the highest number of Instagram followers (20,500). Additionally, the French, Hellenic, and Turkish neurosurgery societies had over 2800, 1800, and 850 followers on Facebook, respectively.

It appears from these results that the utilization of SoMe in Europe is much lower than in North America. In 2016, Alotaibi et al. [3] showed that 18.4% of neurosurgical programs in North America had an affiliated social media account, representing almost a threefold higher adoption 5 years ago. However, this list of programs was limited to academic training programs accredited by ACGME and RCPSC and may therefore not be directly comparable to the list of centers listed on the EANS website.

While language may be a factor limiting the adoption of social media in non-English native speaking countries, other reasons for the discrepancy between North America and Europe may involve concerns regarding privacy and general differences in SoMe culture, as well as less interest from a “marketing” point of view considering differences in health care systems. Laws and regulations such as (HIPAA/GDPR) between countries may also play a role in the capacity of SoMe adoption due to potential content restrictions and legal liability. Besides these possibilities, it is also plausible that SoMe usage differs considerably in Europe amongst individuals and organizations, with elevated SoMe presence potentially amongst individuals, however, this has not been thoroughly investigated. Ultimately, the lack of SoMe engagements from academic programs in Europe is probably due to multiple factors: they require resources, time, competence, high language proficiency, and a SoMe strategy. Neurosurgery departments, unlike most other specialties, typically comprised a small team, and therefore this may be part of the problem; however, the increased utilization of SoMe in North American neurosurgery programs [2] indicates that some form of SoMe presence is achievable.

In terms of neurosurgical journals, besides *JNNP*, *Acta Neurochirugica*, the *Journal of Neurological Sciences* and *Journal of Neurological Surgery Part B* also had active SoMe presence. In particular *Journal of Neurological Surgery Part B* had one of only 2 accounts found on YouTube, and had a relatively large subscriber base and nearly 90,000 views.

### The increasing adoption and importance of SoMe

Studies from various specialties have shown that social media engagement correlated in improved exposure of research work and can influence citations [3, 16, 18]. Direct evidence of this impact was recently supported by a randomized trial which assessed the impact of tweeting or not tweeting, on citations and Almetric score at 1 year, showing that independent predictors of citations included tweeting and exposure to a larger number of Twitter followers [10].

Use of SoMe remains diverse, forms the perspective of patients and neurosurgeons, and from the perspective of geography, and social media platforms. This is exemplified by a recent systematic review of social media in neurosurgery which showed that patients and caregivers most commonly used Facebook and Twitter, whereas nearly 50% of neurosurgeons used LinkedIn and Doximity [13].

While it has become well accepted that SoMe engagement provides multiple beneficial benefits to organizations, by way of branding, public engagement, promoting research and information dissemination, the recent COVID pandemic has also shown that a SoMe presence is critical in continuing conversations between organizations and their stakeholders during times of crises. The cancellations of congresses and changes to online formats including webinars or virtual meetings such as the EANS 2020 meeting (www.eans.org/page/EANS2020_Congress), have demonstrated how critical SoMe use can be. SoMe use during such congresses does not only allow for promoting such events but also is used for Q&A (question and answer) sessions for live presentations. Likewise, COVID-19 also has changed the neurosurgery residency matching process in the USA which normally involves many in-person interviews by candidates who fly across the country and which have been largely changed to video interviews [1]. As a consequence of this, it has been notable that neurosurgery residency programs have made use of their SoMe accounts to engage with potential candidates.

It is important to note that social media provides many potential benefits, but also can bring challenges once they become highly integrated and present with a large following. A large SoMe following may result in increased communication with the institution from the neurosurgery community, patients, and activists, which will require active moderating. Management of this may eventually require input from a media relations team, often from the hospital.

### Opportunities and steps that can be taken to bridge the SoMe gap

There appears to be growing evidence that there are benefits of SoMe for neurosurgery, and it is evident that from a European point of view, further work is necessary to integrate this into our specialty. The EANS, serving as the principal vehicle for association among the various neurosurgical stakeholders, is uniquely positioned to encourage such a transition. Tools for building a SoMe profile have been developed by Neurosurgery Research and Education Fund and may help those interested in creating a SoMe account (Twitter) [11]. While the EANS is increasingly engaging on SoMe platforms, the CNS and AANS have about double and triple the number of followers on Twitter, respectively, which indicated that further growth is possible.

As SoMe in neurosurgery is further developed in Europe, it will also be important to take into consideration areas in particular need of improvement. It has been recently highlighted that dissemination of content via SoMe in the form of videos remains poor, and is an area which could benefit from particular attention [13, 17].

Ultimately, the lack of SoMe adoption indicates that there is an opportunity for growth in this area. Depending on the situation, including financial, human resources and size of the country, efforts can be made to optimize a SoMe strategy. For example, larger institutions may benefit from individualized accounts, whereas smaller institutions could seek to develop their neurosurgical society SoMe presence, as this remains an area of need in development as well. Increased, SoMe development can facilitate communication of neurosurgical departments, particularly smaller ones, with a global audience.

## Limitations

As SoMe usage changes on a daily basis, the number of followers as well as other SoMe parameters may differ from those reported. Additionally, since the search and writing of this paper, it is possible that new accounts have been created which are not included in the present paper. It is also possible that some accounts were missed, despite attempts to find all relevant accounts. However, it is likely that those accounts are either not actively used or do not yet have significant SoMe activity. The search of SoMe accounts for neurosurgery departments was limited to Twitter, Instagram, and Facebook as these are typically more commonly used for larger audiences. LinkedIn is a popular SoMe platform; however, it was not assessed for a number of reasons: (1) principally used by individuals and not departments, (2) profiles may be public or private, (3) usage is typically for connecting with individuals rather than dissemination openly to wide audiences, (4) the large number of profiles would make it unfeasible to accurately assess LinkedIn usage.

## Conclusion

The use of SoMe in neurosurgery in Europe is limited, and no accounts were found in most countries with some notable exceptions. Likewise, SoMe use remains sparse among European neurosurgery journals. Given that SoMe has been seen as an important tool to engage with patients and to correlate with effective dissemination of academic production in other specialties as well as neurosurgery within North America, a concerted effort should be made to promote its uptake among EANS member states.
